# Therapeutic Potential of Stem Cells in Follicle Regeneration

**DOI:** 10.1155/2018/1049641

**Published:** 2018-08-05

**Authors:** Agnieszka Owczarczyk-Saczonek, Magdalena Krajewska-Włodarczyk, Anna Kruszewska, Łukasz Banasiak, Waldemar Placek, Wojciech Maksymowicz, Joanna Wojtkiewicz

**Affiliations:** ^1^Department of Dermatology, Sexually Transmitted Diseases and Clinical Immunology, University of Warmia and Mazury in Olsztyn, Olsztyn, Poland; ^2^Department of Rheumatology, University of Warmia and Mazury in Olsztyn, Olsztyn, Poland; ^3^Department of Plastic, Reconstructive and Aesthetic Surgery, L. Rydygier Collegium Medicum in Bydgoszcz, Nicolaus Copernicus University in Torun, Toruń, Poland; ^4^Department of Neurology and Neurosurgery, Faculty of Medical Sciences, University of Warmia and Mazury in Olsztyn, Olsztyn, Poland; ^5^Foundation for Nerve Cell Regeneration, University of Warmia and Mazury in Olsztyn, Olsztyn, Poland; ^6^Department of Pathophysiology, Faculty of Medical Sciences, University of Warmia and Mazury in Olsztyn, Olsztyn, Poland; ^7^Laboratory for Regenerative Medicine, Faculty of Medical Sciences, University of Warmia and Mazury, Olsztyn, Poland

## Abstract

Alopecia is caused by a variety of factors which affect the hair cycle and decrease stem cell activity and hair follicle regeneration capability. This process causes lower self-acceptance, which may result in depression and anxiety. However, an early onset of androgenic alopecia is associated with an increased incidence of the metabolic syndrome and an increased risk of the cardiac ischaemic disease. The ubiquity of alopecia provides an encouragement to seek new, more effective therapies aimed at hair follicle regeneration and neoregeneration. We know that stem cells can be used to regenerate hair in several therapeutic strategies: reversing the pathological mechanisms which contribute to hair loss, regeneration of complete hair follicles from their parts, and neogenesis of hair follicles from a stem cell culture with isolated cells or tissue engineering. Hair transplant has become a conventional treatment technique in androgenic alopecia (micrografts). Although an autologous transplant is regarded as the gold standard, its usability is limited, because of both a limited amount of material and a reduced viability of cells obtained in this way. The new therapeutic options are adipose-derived stem cells and stem cells from Wharton's jelly. They seem an ideal cell population for use in regenerative medicine because of the absence of immunogenic properties and their ease of obtainment, multipotential character, ease of differentiating into various cell lines, and considerable potential for angiogenesis. In this article, we presented advantages and limitations of using these types of cells in alopecia treatment.

## 1. Introduction

Hair loss is caused by a variety of factors: hereditary (trichodystrophy, androgenic alopecia), concomitant medical conditions, hormonal disorders (thyroid gland disorders, insulin resistance), autoimmune (patchy alopecia, systemic lupus erythematosus), nutritional disorders, environmental factors (medicines, UV radiation), psychological factors (stress, trichotillomania), and ageing. The damaging factors affect the hair cycle and decrease stem cell activity and hair follicle regeneration capability.

Alopecia is commonly regarded as a defect with apparently no significant health consequences. However, hair loss affects self-acceptance, which may result in depression and anxiety [1, 2]. It is not only an aesthetic issue. An early onset of androgenic alopecia is associated with an increased incidence of the metabolic syndrome and an increased risk of the cardiac ischaemic disease [3]. The ubiquity of alopecia provides an encouragement to seek new, more effective therapies aimed at hair follicle regeneration and neoregeneration.

### 1.1. Stem Cells in the Hair Follicle

**Figure 1:**
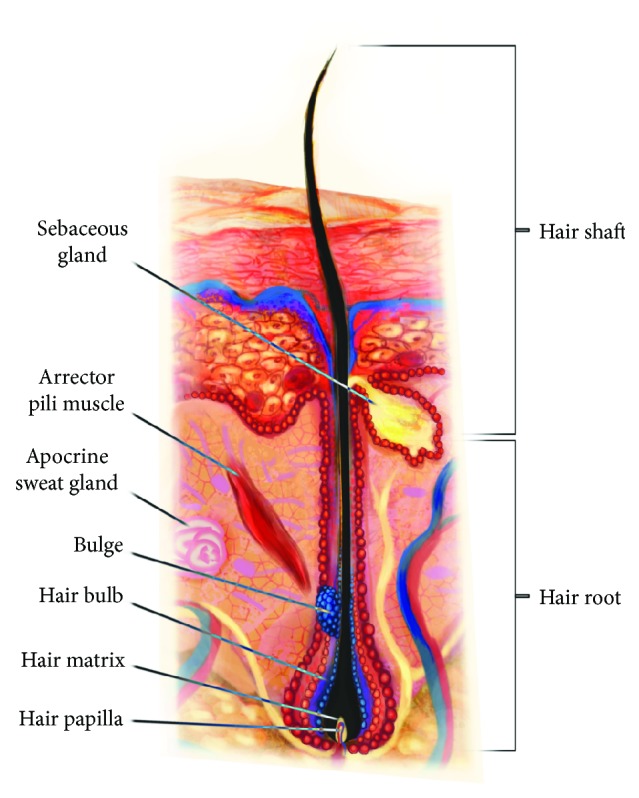
Hair follicle anatomy.

Hair follicles have a niche for mature stem cells—hair follicular stem cells (HFSCs)—a so-called “bulge” in the attachment region of arrector pili muscles, which contain epithelial and melanocyte stem cells. Moreover, HFSCs are also situated within the outer root sheath (ORS), within the region of the proximal end of the isthmus—this area is also known as the “bulge” [4]. HFSCs take part in the regeneration of epidermal cells and the structure of hair follicles and sebaceous glands [5] (Figure 1).

Stem cells of the “bulge” can remain in their niche where they self-regenerate, but they can also move down to the hair matrix region, where they become progenitor cells which then form an internal hair follicle and the hair stem [6]. The “bulge” region is not uniform, with two compartments distinguishable in it: the lower part, close to the hair matrix, which generates the internal hair follicle cell line, and the upper part, which self-regenerates, but which does not directly participate in the regeneration of the hair follicle [7, 8]. Since the heterogeneity of the “bulge” also depends on its relationship with the basal membrane, two populations of CD34+ cells are distinguished. One of them, the so-called suprabasal SCs, contains lower levels of a6-intergin and has a lower proliferative potential [6, 9]. The “isthmus” region is, apart from the “bulge,” another one which also contains stem cells participating in the formation of interfollicular epidermis and sebaceous glands [7, 10].

Another type of stem cells within the hair follicle is dermal papilla cells (DPCs), probably originating from dermal condensation, which is the initial stage of the hair follicle development [11, 12]. DPCs play an important role in induction and regulation of hair growth and the formation of new hair follicles [11, 13, 14]. Signals from DPCs activate stem cells in the “bulge” and germinal matrix cells in the late telogen/early anagen phase [11, 15] by activating the Wnt/*β*-catenin pathway [11, 16]. Moreover, DPCs have potential for differentiation into lines of adipocytes and osteocytes [11, 17], and they can be transformed into pluripotential cells [11, 18].

Alopecia involves changes in two types of hair stem cells, both human hair follicle stem cells (HFSCs) and dermal papilla cells (DPCs) [19, 20]. They ensure conditions for proper hair regeneration [20]. In scarring alopecia (lupus erythematosus, lichen planus), inflammatory cell infiltration around the bulge results in an irreversible loss of HFSCs. Although the progenitor cells are damaged, HFSCs are preserved in patchy and androgenic alopecia. This is why this type of alopecia can be reversible [20].

Stem cells of the “bulge” are increasingly well characterised, especially in murine hair follicle, which facilitates their identification, although no universal marker has been found for them. One of them is cytokeratin 15 (CK15), which is why CK15+/integrin *α*6+ or CD34+/integrin α6+ cells have been identified as “bulge” cells [21]. Studies on murine hair follicles have also revealed expression of, inter alia, CK19 [8, 22] and numerous transcription factors, that is, Sox9, Lgr5, Gli1, Hopx, LHX2, Nfatc1, and Tcf3 [8, 25]. However, expression of certain markers depends on the hair cycle phase and on the precise location of the cells within the bulge [7, 8]. Lgr5, a receptor involved in the Wnt signaling pathway, has been identified as an actual marker of the hair follicle stem cells [25]. Stem cells of the upper and lower parts of the bulge in the telogen hair follicle affect the expression of CD34 and only of the lower part of Lgr5. Cells participating in the formation of a new anagen hair express Lgr5, but not CD34 [26]. Cells of the upper part of the “bulge” present a higher expression Nfatc1, which is associated with a state of rest [6]. Expression of Lgr6 [7, 10] and Lrig1 [7, 27] has been observed within the “isthmus.” Meanwhile, progenitor cells of the germinal matrix are derived from stem cells of the bulge but, unlike them, exhibit a high level of P-cadherin [8, 28].

Human hair follicle stem cells are less known than murine HFSCs. It seems that certain markers are common to both human and mouse HFSCs, that is, CD34 [4, 23], K15 [4, 19], K19 [4, 29], and CD200 [4, 19, 23]. The presence of other markers, i.e., Sox9 and LHX2, requires further studies [30]. Markers found only in human stem cell follicles belong to PHLDA1 [4, 24] and EpCAM/Ber-EP4, which is a useful marker of the telogen secondary hair germ [4, 28].

Dermal papilla cells present different markers, including those from hair follicle cells and dermal fibroblasts [11]. Alkaline phosphatase (ALP) is the most important for both human and murine HFs and is the most specific of the markers [11, 29, 30]; its high activity is a marker of DP cell differentiation [11, 31]. Moreover, expression of *α*-SMA [11, 17], laminin, and fibronectin [11] as well as CD133 [11, 32] has been observed in DPCs.

Marker expression changes in pathological states. Immunoreactivity of CK15 is decreased in people with patchy alopecia, and it is present in androgenic alopecia [21]. Hair follicles in the frontal parts of the scalp exhibit a deficit of CD34 in androgenic alopecia, and its expression is preserved in hair follicles of the occipital region [21]. Another marker CD200 of matrix cells is poorly expressed in patchy alopecia, which may be a sign of the disappearance of the immune privilege and can contribute to pathogenesis (reaction of autoreactive lymphocytes) [21, 33].

Stem cells in the bulge remain in the resting phase for most of their lives, but they can be activated depending on the hair cycle phase. Most of the concepts regarding the course and regulation of the hair cycle have been created during research on mouse models. During the hair cycle in mice, in the anagen phase, stem cells in the bulge are divided three times on average and stay within the niche, whereas cells of the germinal matrix divide intensely and differentiate, forming the growing hair stem. During the catagen phase, cells of the germinal matrix undergo apoptosis; stem cells of the bulge migrate out of it to the external hair follicle, and subsequently, at the end of the catagen phase, they form a new bulge around the hair stem and a new germinal matrix under the bulge. Stem cells in the bulge remain in the state of rest during the telogen phase, and between the telogen and anagen phases, they self-regenerate or migrate, creating a pool of germinal matrix cells which subsequently proliferate to form the hair matrix [6]. The precedence has been shown for the derivative cells in the bulge, the so-called “SC” progenitor cells of the germinal matrix, in the expression of genes that affect stem cell activation, and precedence in proliferation during the regeneration cycle, even before the cells of the bulge [8, 15, 34]. The translation of the mouse hair cycle into the human hair cycle has some limitations due to the different lengths of anagen [35, 36], asynchrony of the human cycle [35, 37], or a different reaction to the influence of hormonal factors [35, 38]. Currently, studies are conducted on human scalp skin xenografted into immunocompromised mice to establish the course of the hair cycle in vivo in humans [35].

The activity of stem cells in the bulge is controlled by the microenvironment that surrounds it, a so-called “niche.” This includes daughter cells of stem cells of the bulge, which activate their self-regeneration during early and late anagen phases [39].

Stem cells are significantly affected by mesenchymal cells of the dermal papilla, which are in close contact with cells of the germinal matrix, separated only by the basal membrane [7]. They seem to be of key importance in the induction of hair growth and in signal transmission during its regeneration [8, 34]. Experiments have shown that hair regeneration is not possible after laser ablation because the hair follicle cycle stops at the telogen phase without progressing to the anagen phase [6, 7, 34, 40]. Injections of exosomes derived from DPCs to HFs have been found to accelerate the entry of anagen and catagen delay via the *β*-catenin and Shh pathways [41]. HFSCs are also affected by fibroblasts in the reticular and papillary layers of the dermis as well as of the subcutaneous tissue [7].

Within the niche there are also melanocyte stem cells, which are responsible for the formation of mature melanocytes that impart the colour to a growing hair. The survival and growth of MSCs depend on signals transmitted by hair follicle epithelial cells, for example, the TGF-*β* or the Wnt pathway [7, 39]. The extracellular matrix is another component of the microenvironment. It directly affects stem cells by the formation of the basal membrane, with which stem cells are in contact modulated, for example, by integrins [6, 8].

Stem cells of hair follicles are also affected by the macroenvironment surrounding hair follicles, for example, adipose tissue. It seems to undergo similar changes to those of the hair follicle: the thickness of the adipose tissue increases during the anagen phase, and adipocytes proliferate intensively [8, 42]. Adipocytes secrete BMP2 during the late catagen phase and early telogen phase, which favours the resting states in the niche, whereas secretion of BMP2 is reduced at the end of the telogen phase, which supports the activation of HFSCs [8, 42, 43]. Communication between adipose tissue and the epithelium runs in both directions. Mutations blocking the hair cycle have been found to inhibit adipogenesis, which suggests that epithelium cells send signals activating the proliferation of adipocytes [6, 42].

Nerve ends affect stem cells situated at the upper part of the Gli1+ bulge by a signal of the Shh pathway [6, 44]. Therefore, denervation can reduce the effect of SCs in the “isthmus” on wound healing [6, 44]. However, it seems that nerves are not indispensable components of the niche, because denervation does not impair hair follicle regeneration, which may suggest that Gli+ cells receive Shh signal from other sources [7].

The hair follicle absorbs nutrients from the surrounding microvascular network, which is transformed during the hair cycle—angiogenesis is increased during the anagen phase [6, 45]. Cells of the bulge and of the matrix can probably stimulate angiogenesis [6]. Delayed induction of angiogenesis, which accompanies impaired angiogenesis, has been observed in mice [7, 45]. It has been suggested that stem cells in general prefer a low-oxygen environment, where they secrete marker of hypoxia [6, 46]. The vascular network, especially that surrounding the “isthmus,” containing venous vessels, can participate in maintaining the low-oxygen environment in the surrounding of the stem cell environment [6].

**Figure 2:**
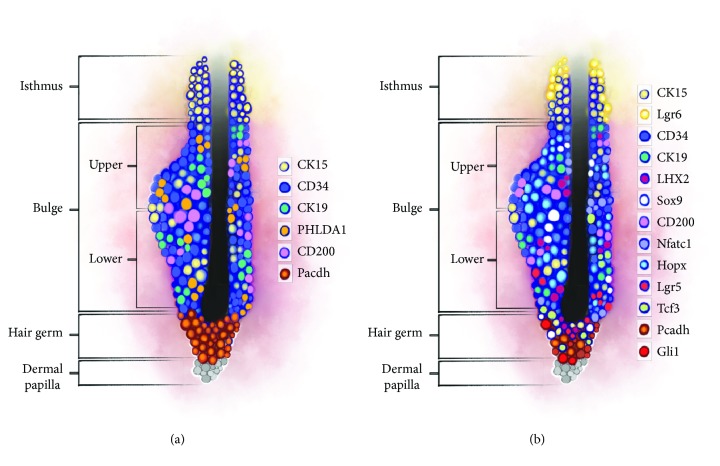
The markers of hair follicle: (a) in human, (b) in mouse.

However, although the effect of the immune response has not been sufficiently elucidated, it is important that the role of maintaining the immune privilege of hair follicles, associated with decreased expression of MHC I molecules and with increased secretion of local immunosuppressors, should be maintained during the anagen phase [6, 47]. The loss of this privilege and an immune attack on cells of the matrix and the bulge are associated with alopecia [6, 48]. Dermal cells *γδ*T are known to modulate posttraumatic regeneration of hair follicles by secreting FGF9 [7, 49]. Macrophages, in turn, increase the level of Wnt7b and Wnt10a ligands during the telogen phase after undergoing apoptosis, whereby activating HFSCs [6, 7, 50]. Macrophages play an important role in posttraumatic activation of HFSCs—arresting their recruitment into the wound delays hair growth, whereas transplantation of active macrophages is sufficient for induction of hair growth [6, 51]. Also important is the role of Treg, which presents a high level of Jag 1 from the Notch family, which affects the effective regeneration of HF [52] (Figure 2 and Table 1).

### 1.2. Stem Cell Use in Hair Follicle Regeneration

Hair follicles are immunologically privileged places, like the brain, eyes, and testicles, and they are under the influence of the neuroendocrine-immune network [32]. In physiological conditions, this is affected by

1. low expression or absence of the main MHC I antigens,
2. the presence of malfunctional Langerhans cells,
3. local expression of immunosuppressive substances (TGF-*β*1 and *α*-melanocytes MSH) [32, 48]. Owing to this, they can be easily used in transplantation.

Multipotent stem cells can regenerate hair follicles with sebaceous glands in the skin. In the current state of knowledge, stem cells can be used to regenerate hair in several therapeutic strategies:

1. Reversing the pathological mechanisms which contribute to hair loss (especially in androgenic alopecia)
2. Regeneration of complete hair follicles from their parts (cells in the bulge can regenerate a whole hair)
3. Neogenesis of hair follicles from a stem cell culture with isolated cells or tissue engineering [5, 53, 54]

### 1.3. Studies of Use of Autologous Stem Cell in Hair Follicle Regeneration

Hair transplant has become a conventional treatment technique in androgenic alopecia (micrografts, follicular unit transplantation (both FUT an FUE), and individual follicular group harvesting (IFGH)) [55]. Although an autologous transplant is regarded as the gold standard, its usability is limited, because of both a limited amount of material and a reduced viability of cells obtained in this way. Currently, methods are being developed which enhance the effectiveness of the use of autologous stem cells of the hair follicle.

Apart from cells of the “bulge,” stem cells reside specifically in the HF mesenchyme and function to replenish the dermal papilla and connective tissue sheath. They are called self-renewing dermal stem cells (DSCs) [56, 57]. When transplanted, DSCs integrate with mesenchymal cells and they act together with epithelial stem cells, participating in creating new hair follicles [56, 57]. In cultures, they form spherical, self-regenerating colonies. However, it is labour-consuming and ineffective. Therefore, methods have been developed for their simultaneous collection, isolation, and administration *in vivo* at acceptor sites with the use of the so-called stirred suspension bioreactors. They help to obtain cells of greater uniformity; increased cell density per volume; and control of the concentration of nutrients, metabolites, and growth factors [56, 58].

The findings of the study by Agabalyan et al. have confirmed that cells can retain their phenotype and an ability to form hair follicles even after five passages in bioreactors. Moreover, the productivity is five times higher compared to static cultures [56]. This has given rise to the possibility of using this method commercially in the treatment of alopecia.

Gentile et al. [5] demonstrated the application of an innovative Rigeneracons® bioreactor (certificate CE, class I) in order to provide autologous micrografts and their immediate use in clinical practice. They proved that cells isolated from the bulge region can improve the thickness of hair in patients affected by androgenic alopecia using a new method of isolating human mature stem cells obtained from a patient self-biopsy, without culturing. Enhanced hair thickness was achieved in 11 men (aged 38 to 61 years) with androgenic alopecia even at the stage of 3–5 in the Norwood-Hamilton scale. After the biopsy and removal of unwanted remnants of fatty tissue, a medical device Rigeneracons (certificate CE, class I) was used to obtain cell suspension. The percentage of mesenchymal stem cells CD44+ from the dermal papilla was approx. 5% + 0.7% and CD200+ from the bulge was approx. 2.6% + 0.3%. After 23 weeks of therapy, after the last administration of stem cells, the average number of hairs and their thicknesses increased by 29% ± 5% compared to the baseline for the hair thickness in the treated area and by less than 1% of hair thickness increase in the placebo area [5].

Furthermore, Nilforoushzadeh et al. [59] evaluated the regeneration potential of cultured mature dermal papilla to induce the growth of a hair follicle injected to the skin of bare mice. Initially, dermal papilla cells in the culture were observed to multiply with expression of CD200, and these fusiform cells tended to form colonies after three to five days. Subsequently, after two weeks, they acquired a passaging capability and they formed an extracellular matrix after the third passaging. Histopathological examination in mice which received 1.2 × 10^6^ of cells of dermal papilla revealed structures that transformed into hair follicles at sites of injection in the dermis [59].

Ibrahim et al. used autologous bone marrow mononuclear cells (BMMC) (including stem cells) to treat refractory patchy alopecia and androgenic alopecia, and the therapeutic effects were compared to the group treated with autologous stem cells of hair follicles. Cells were administered in a single application (1 millilitre in a density of 100,000 cell/ml was injected, using a 26-gauge needle, intradermally at per centimetre square of the treated site), and a significant improvement was observed in all patient groups under treatment [21]. Interestingly, the effect of stem cells was similar despite the fact that they had been obtained from two different sources. The effect of intradermal injection of BMMC may result from the diversity of the cell mixture: progenitor, hematopoietic stem cells, various types of inflammatory cells, and mesenchymal stem cells. BMMC can stimulate hair growth as a consequence of the ability to differentiate into various cell types, the ability to secrete bioactive molecules which stimulate angiogenesis (VEGF) and anti-inflammatory molecules with an immunomodulatory and antiapoptotic effect [21].

### 1.4. Studies of the Use of Adipose-Derived Stem Cells

ADSCs (adipose-derived stem cells) seem an ideal cell population for use in regenerative medicine because of the absence of immunogenic properties, their ease of obtainment, multipotential character, ease of differentiating into various cell lines, and considerable potential for angiogenesis. ADSCs have been shown to originate from mural cells located in the perivascular niche, vascular smooth muscle cells and pericytes, both involved in the formation of normal vasculature and are responsive to VEGF [60]. Naturally, hair follicles surrounded by subcutaneous adipose cells and by dermis form an interfollicular dermal macroenvironment, which is important in maintaining the proper growth of bulge and follicle cells [11, 61, 62]. ADSCs are indispensable in the activation of epidermal stem cells, which they do by secreting growth factors. The vascular endothelial growth factor (VEGF) regulates hair growth and the size of the hair follicle size by stimulation of angiogenesis. The hepatocyte growth factor (HGF) is involved in the duration of the hair cycle phases. The platelet-derived growth factor induces and maintains the anagen phase, and the insulin-like growth factor I (IGF-I) controls the hair growth cycle and hair cell differentiation [11, 63–67]. Another direction of their action is the stimulation of angiogenesis and an improvement of the blood supply to dermal papilla cells. They also have immunomodulatory and immunosuppressive properties through the direct interaction between cells and secretion of prostaglandin E2 (PGE2), leukaemia-inhibiting factor (LIF), and kynurenine [11, 62].

The paracrine activity of ADSCs is highly complex, and the factors secreted by stem cells have both a direct and an indirect effect on hair follicles. TB4 contributes to the activation of stem cells in a hair follicle, increasing their migration into the follicle and differentiation. SDF-1 acts through an increase in expression of EGR-1; it also increases the cell tropism towards the follicle and increases angiogenesis. The action of MCP-1 is less obvious; despite being an inflammatory factor, it has a proven tissue regenerative effect; also, a significant role of the microenvironment in the effect of paracrine factors in promoting the growth of the hair follicle has been emphasised [68]. Huang et al., in a study on rats, found that an addition of ADSCs to a culture of dermal papilla cells or core cells, the inner and outer sheath, enhances their viability [64]. A significant increase in the regenerative potential was recorded in a study by Huang et al., in which ADSCs were enriched with LL-37, which is an antibacterial peptide occurring naturally in wounds [64]. That study showed a significant increase in the local regenerative factors (endothelial growth factor, thymosin beta-4, monocyte chemoattractant protein-1, and stromal cell-derived factor-1). A significant promotion of the growth of hair follicles, in both *in vitro* and *in vivo* animal models, was observed [60, 69–71].

Physiologically, adipose tissue surrounding hair follicles plays an important role in extending the anagen phase. Adipocytes progenitor cells have been observed to multiply during the transition from the telogen to the anagen phase, around the hair follicle [61, 64]. The thickness of the subcutaneous layer increases significantly during the intense hair growth phase (anagen) compared to their amount in the resting phase (telogen) [11, 59]. ADSCs stimulate hair follicle cells through peroxisome proliferator-activated receptors, whose three isoforms have been found on their surface (PPAR*α*, PPAR*γ*, and PPAR*δ*) [64]. Meanwhile, mature adipocytes have a negative effect on hair follicle cell proliferation and on proliferation of fibroblasts surrounding the hair follicle in simultaneous culture systems [11, 72].

Interestingly, a change in adipocyte cell line properties can cause skin and hair disorders. Lipid metabolism disorders can cause defects in the skin structure and functions. Over-expression of human apolipoprotein C1 (APOC1) with hyperlipidemia in transgenic mice causes hair growth disorders correlated with the level of expression of human APOC1 gene in the skin [11, 73].

Hypoxia, which is not toxic to mesenchymal cells, also increases the production of growth factors for ADSCs: vascular endothelial growth factor (VEGF), platelet-derived growth factor (PDGF), hepatocyte growth factor (HGF), and insulin-like growth factor II (IGF-II) [74, 75]. The effect of hypoxia on ADSCs was examined in a study conducted by Park et al. [74]. ADSCs passaged four times with CO_2_ subsequently administered subcutaneously to mice to observe induction of the anagen phase and proliferation of human follicular cells of the dermal papilla and keratinocytes. Hypoxia resulted in increased secretion of insulin-like growth factor-binding protein-1 and protein-2 (IGFBP), macrophage colony stimulating factor (M-CSF), M-CSF receptor, PDGF receptor-*β*, and VEGF, whereas secretion of the epidermal growth factor was smaller [74].

Unfortunately, two-dimensional (2D) cultures of dermal papilla cells lose their hair formation capability in culture, which is why they require maintaining their spheroidal forms (3D) [76, 77]. It is a challenge to develop methods that mimic *in vivo* conditions, which both maintain the 3D structure of cells and contain a special medium which imitates a natural niche rich in growth factors [56, 75].

Currently, there are no known tissue regeneration protocols applied for hair transplant with the use of ADSCs. Zanzoterra et al. [78] examined the capabilities of autologous cell suspension in the Rigenera system, which were obtained by mechanical fragmentation of subcutaneous and adipose tissue from the occipital area. The cell suspension was applied in the hair transplant area, thereby increasing the amount of growth factors. Microdamage has been observed to heal more quickly and to transplant hair to grow continuously even two months after the procedure, with the telogen phase shortened [78].

An ADSC-conditioned medium (ADSC-CM) was used in patients with alopecia (both male and female) in a study by Fukuoka and Suga [63]. A commercial product containing a protein solution with ADSCs was used (AAPE, Prostemics, Seoul, Korea) with various growth factors (hepatocyte growth factor, fibroblast growth factor I, granulocyte colony-stimulating factor, granulocyte macrophage-colony-stimulating factor, IL-6, VEGF, and TGF). The preparation (0.02 ml/cm^2^ of the solution) was administered intradermally every 3 to 5 weeks (4–6 sessions), and hair growth was monitored with trichograms. A significant improvement in hair thickness was achieved in patients of both sexes [63].

Shin et al. [71] used ADSC and conditioned media of ADSCs (ADSC-CM) in a retrospective, observational study in 27 women with a female pattern hair loss (FPHL). The application of ADSC-CM showed efficacy in treating FPHL after 12 weeks of therapy with increased hair density and thickness without severe adverse reactions [71]. Won et al. [79] also showed that the application of ADSC-CM enhanced proliferation of cultured human dermal papilla cells (DPCs) by up to 130% [79].

Other studies have confirmed that enriching adipose tissue with a stromal vesicular fraction (SVF) supports adipocyte viability and yields better outcomes for a hair transplant procedure when they are present in grafts [62, 80]. Lipoaspirate obtained from abdominal fat (system Puregraft LLC, Solana Beach, CA, USA) was administered to the scalp at 1.0 ml/cm^2^ in a Perez-Meza study. The amount of hair was found to increase by 23% after six months of the follow-up period [80].

### 1.5. Studies on Using Stem Cells from Wharton's Jelly

#### 1.5.1. The Advantage of Stem Cells from Wharton's Jelly Compared to Other Mesenchymal Cells

Wharton's jelly has become a preferential source of stem cells due to its ready availability from a large pool of donors, noninvasive and painless acquisition, no risk to the donor, no ethical limitations, weak immunogenic potential, and high multipotential differentiation capability [81, 82]. Moreover, exposure to infectious agents occurs rarely, which guarantees safety to the donor [83].

Additionally, the decellularized Wharton's jelly matrix (DWJM) (fresh jelly was subjected to two cycles of osmotic shock, alternately with a hypertonic solution of NaCl, mannitol, MgCl_2_, and KCl with the osmolarity of approx. 1.275 mOsm/l, and centrifuged at 5000 rpm at 4°C against a hypotonic solution of 0.005% Triton X-100) can provide a natural scaffolding for stem cells as a biocompatible matrix, which supports their viability, initiating aggregation of mesenchymal cells. DWJM contains TGF-*β*, collagen I, fibronectin, and tenascin, which may be responsible for condensation of added WJMSC in some areas of the DWJM. Hence, DWJM is a natural biocompatible 3D matrix which ensures adhesion, penetration, growth, and proliferation of cells—both *in vitro* and *in vivo*. To conclude, this paper presents DWJM as a new and natural 3D scaffolding which can be used in tissue engineering and regenerative medicine [84].

#### 1.5.2. Neogenesis of Hair Follicles with Stem Cell Culture on Media and Grafting Them into the Skin: *In Vitro* Regeneration

In 2013, two researchers, demonstrated that it is possible to obtain cells with an expression of cytokeratin 19 and hair-like structures from WJMSC in *in vitro* conditions. Cytokeratin 19 (CK19) is a marker of bulge stem cells which determines the self-regeneration potential of modified skin [85, 86].

The Korean team of Yoo et al. [87–89] examined the effect of hWJSC on the acceleration of wound healing processes along with formation of hair follicles and other skin appendages. Enriched aggregates of hWJSC cells can form new hair follicles. The addition of growth factors to the culture medium is required: hepatocyte growth factor (HGF) stimulates growth of hair follicles *in vivo* and *in vitro*; basic fibroblast growth factor (bFGF) stimulates growth of dermal papilla cells *in vivo*; and vesicular endothelial growth factor (VEGF) stimulates growth of hair follicles and hair root *in vivo*. The hepatocyte growth factor (HGF) must be used at the stage of differentiation of the dermal papilla in culture, which is relatively expensive [87–89].

Moreover, Yoo et al. [88] compared effects of culturing bone marrow and umbilical cord stem cells to the spontaneous formation of dermal papilla-like tissues (DPLT). Isolated cells of the hair outer sheath were used for incubation: DPLT were recovered from 25T cell culturing plates and mixed with 1106 cells of hair sheath in 50 ml of physiological saline and injected into mice skin. The mice were examined after six weeks. Subsequently, the clinical effects of hair follicle formation in originally bare mice following their implantation in the skin were compared. No differences between the methods were observed [88].

Wu et al. (a Chinese study) [90] demonstrated the potential for differentiation of hMSC (from human embryos) to dermal papilla cells in cocultures of hMSC using dermal papilla cells previously obtained from patients. Expression of versican, CD133, SCF (stem cell factor), ET-1 (endothelin-1), and bFGF (fibroblast growth factor) was observed during the process of differentiation [90].

#### 1.5.3. Neogenesis of Hair Follicles from Isolated Cells (Stem Cells from Wharton's Jelly): *In Vivo* Regeneration

Intensive studies are being conducted on the commercial use of hWJSC in alopecia treatment at the University of Kansas Innovation and Collaboration, Kansas, USA (Dr. Omar Aljitawi) (no literature data) (Tables 2 and 3).

## 2. Conclusion

Maintaining a pool of stem cells is necessary for tissue homeostasis and damage repair. Their divisions are not frequent in mature organisms, and most of them are in a dormant state. Therefore, it is important to understand the mechanisms of their activation, which will allow for the use of multipotent cells in regenerative medicine [33]. Their use is additionally complicated by the fact that expression of receptors on different growth factors and the effect of the microenvironment may vary. Moreover, not all target points in stem cell therapy have been identified. It requires further studies aimed not only at the use of stem cells and their various fractions and compositions with adjuvants but also at broadening of knowledge on the physiology and cytophysiology of the hair follicle [35].

## Figures and Tables

**Table 1:** The markers of hair follicle and their role.

Author	Signal	Researched object	Conclusions
Telerman et al., 2017 [91]	Blimp1	Transgenic mouse	Ablation delayed HF morphogenesis, and growth and prevented new HF formation after wounding; role in promoting the dermal papilla inductive signaling cascade that initiates HF growth
Kobielak et al., 2007 [92]	Bmpr1a	Transgenic mouse	Ablation leads quiescent SCs to activate and to proliferate, causing an expansion of the niche and loss of slow-cycling cells; HFSCs are unable to terminally differentiate into hair
Lei et al., 2014 [93]	DKK1	Transgenic mouse	DKK reduce hair follicle enlargement and decrease proliferation; injection of DKK1 during early anagen significantly reduced the width of prospective hairs
Millar et al., 1999 [94]	Dvl2	Transgenic mouse	Overexpression in the outer root sheath causes the short-hair phenotype
Lin et al., 2015 [95]	FGF1, FGF2, FGF10	Transgenic mouse	Topical application of FGFs induced an earlier anagen phase and prolonged the mature anagen phase; FGFs promoted hair growth by inducing the anagen phase in telogenic mice
Kimura-Ueki et al., 2012 [96]	FGF18	Transgenic mouse	Ablation causes telogen to become very short, giving rise to a strikingly rapid succession of hair cycles
Higgins et al., 2014 [97]	FGF5	DNA from families with long eyelashes	FGF5 is associated with long-hair phenotype
Guo et al., 1993 [98]	FGF7	Transgenic mouse	Overexpression causes marked suppression of hair follicle morphogenesis
Petiot et al., 2003 [99]	Fgfr2	Transgenic mouse	Lack of Fgfr2 leads to a decreased number of HFs, and follicles were developmentally retarded
Öztürk et al., 2015 [100]	Gab1	Transgenic mouse	Lack of Gab1 caused HF not to enter catagen; instead HFSCs lose quiescence
Mill et al., 2003 [101]	Gli2	Transgenic mouse	Lack of Gli2 causes arrest in HF development with reduced cell proliferation and Shh-responsive gene expression, but normal epidermal differentiation
Estrach et al., 2006 [102]	Jagged-1	Transgenic mouse	Deletion of Jagged-1 results in inhibition of the hair growth cycle and conversion of hair follicles into cysts of cells undergoing interfollicular epidermal differentiation
Amalia Pasolli et al., 2014 [103]	LHX2	Transgenic mouse	Ablation of LHX2 results in cellular disorganization and HFSC polarization within the niche. LHX2 loss leads to a failure to maintain HFSC quiescence and hair anchoring and progressive transformation of the niche into a sebaceous gland
Öztürk et al., 2015 [100]	Mapk	Transgenic mouse	Activation of Mapk signaling can restore quiescence of the SCs
Du et al., 2018 [104]	miR-214	Human scalp skin tissue; in vitro	Downregulation of miR-214 promotes the proliferation and differentiation of HFSCs; overexpression of miR-214 led to decreased expression of EZH2, *β*-catenin, and TCF4
Horsley et al., 2008 [105]	Nfatc1	Transgenic mouse	Ablation causes stem cells to be activated prematurely, resulting in precocious follicular growth
Krieger et al., 2018 [106]	NF-*κ*B	Transgenic mouse	Role in HF stem/progenitor cell activation during anagen induction, involvement in hair fiber morphogenesis during HF cycling
Demehri and Kopan, 2009 [107]	Notch	Transgenic mouse	Absence of Notch signaling leads bulge stem cell descendents to retain their capacity to execute the follicular differentiation program but failing to maintain it owing to their genetic deficiency
Lin et al., 2011 [108]	Pofut1	Transgenic mouse	Disruption of Pofut1 in HF resulted in aberrant telogen morphology, a decrease of bulge SC markers; HF displayed a delay in anagen reentry and dysregulation of proliferation and apoptosis during the hair cycle transition
Oro and Higgins, 2003 [109]	Ptch	Transgenic mouse	Reduced Ptch is associated with tumor formation during anagen
Hoi et al., 2010 [110]	Runx1	Transgenic mouse	Role in promoting anagen onset and HFSC proliferation
St- Jacques et al., 1998 [111]	Shh	Transgenic mouse	Shh signaling is not required for initiating hair follicle development; however, it is essential for controlling ingrowth and morphogenesis of the hair follicle
Kadaja et al., 2014 [112]	Sox9	Transgenic mouse	Sox9-deficient bulge HFSCs begin to differentiate into epidermal cells; as HFSC numbers dwindle, outer root sheath production is not sustained, and HF down-growth arrests prematurely
Foitzik et al., 2000 [113]	TGF-*β*1	Transgenic mouse	Injection of TGF-beta1 induced premature catagen development
Foitzik et al., 1999 [114]	TGF-*β*2	Transgenic mouse	Ablation causes delay of hair follicle morphogenesis, with a 50% reduced number of hair follicles
Oshimori and Fuchs, 2012 [115]	TGF-*β*RII	Transgenic mouse	TGF-*β*2 signaling antagonizes BMP signaling in HFSCs with increased telogen length
Qiu et al., 2017 [116]	TPA	Transgenic mouse	Refractory telogen hair follicles entered anagen prematurely after TPA treatment, with the enhanced proliferation of CD34-positive hair follicle stem cells
Lei et al., 2014 [93]	Wnt10b	Transgenic mouse	Prolonged overexpression increased the size of regenerated hair follicles and increased expression of CD34 in the bulge
Millar et al., 1999 [94]	Wnt3	Transgenic mouse	Overexpression causes a short-hair phenotype and cyclical balding resulting from hair shaft structural defects
Dong et al., 2017 [117]	Wnt7a	Transgenic mouse	Cultured human umbilical cord-MSCs (UC-MSCs) overexpressing Wnt7a can accelerate wound repair and induce hair regeneration via cellular communication in the wound microenvironment
Kandyba and Kobielak, 2013 [118]	Wnt7b	Transgenic mouse	Underexpression causes shorter anagen, premature catagen onset with overall shorter hair production, and diminished HF differentiation marker expression
Enshell-Seijffers et al., 2010 [119]	*β*-Catenin	Transgenic mouse	Inactivation in DP of HF results in reduced proliferation of the progenitors and their immediate progeny that generate the HS and premature catagen

HF: hair follicle; HS: hair shaft; DP: dermal papilla; SC: stem cell.

**Table 2:** Researches on the use of stem cells in the regeneration of hair follicles.

Authors	Research object	Indication	Methods of obtaining	Results	Comments
*Usage of hair follicle stem cell*					
Agabalyan et al., 2016 [56]	Sprague Dawley rats/nude mice	Nude mice genetically mutated	Bioreactors and static cell cultures with bFGF, PGF	Inducing de novo HF formation, reconstituting the DP and connective tissue sheath	Compared with static culture, stirred suspension bioreactors were significantly reduced, but they can generate larger numbers of autologous DSCs, maintaining their regenerative function
Nilforoushzadeh et al., 2016 [59]	Human/mice	Nude mice genetically mutated	Human scalp biopsy, isolation of only papilla cells which were cultured and injected into nude mice	Evidence of hair growth in mice received epithelial and DP cells	The combination of human cultured DP and epithelial cells could induce HF in nude mice
Elmaadawi et al., 2018 [21]	Human	Alopecia areata and androgenetic alopecia	Autologous bone marrow-derived mononuclear cells compared to follicular stems cells (skin punch biopsy from unaffected areas)	Good clinical improvement in both diseases	Nonstatistically significant difference between the source of cells
Gentile et al., 2017 [5]	Human	Androgenetic alopecia	Biopsies were collected and disaggregated by Rigeneracons without culture condition, then injected to the frontal scalp	A 29% ± 5% increase in hair density for the treated area and less than 1% in hair density for the placebo area	No culture required, quick time of surgery (about 60 min)
Kalabusheva et al., 2017 [120]	Human	*In vitro* study	Human DP cells and skin epidermal keratinocytes in a hanging drop culture to develop an artificial HF germ	Aggrecan, biglycan, fibronectin, and hyaluronic acid significantly stimulated cell proliferation in a DP cell monolayer culture without any effect on DP cell identity	Most of the ECM compounds prevented the formation of cell aggregates while hyaluronic acid promoted the formation of larger organoids
Hoffman et al., 2018 [121]	Human/mice	*In vitro* study	Hair follicle-associated-pluripotent stem cells from human scalp skin and transgenic mice with nestin-driven GFP	Intensive hair growth was observed in the pieces of shaved mouse skin histocultured on Gelfoam	Model for chemotherapy-induced alopecia (observing a doxorubicin effect)

*Usage of adipose-derived stem cells*					
Park et al., 2010 [74]	Human/mice	C (3)H/NeH nude mice	ADSCs in a conditioned medium injected subcutaneously induced the anagen phase from telogen and increased hair regeneration in nude mice	ADSCs in a conditioned medium increased the proliferation of human DP and human epithelial keratinocytes; the effect of hypoxia on ADSC function increased hair regrowth	The secretion of IGFBP, M-CSF receptor, PGF, and VEGF was significantly increased by hypoxia, while the secretion of EGF production was decreased
Zanzoterra et al., 2014 [78]	Human	Androgenic alopecia	Injection of ADSCs and growth factors	After 2 weeks, the healing of microwounds was complete and HF continued growing	Rigenera system for the automated mechanical disaggregation of cell population
Sabapathy et al., 2016 [82]	Rats	*In vitro* study	ADSCs isolated from rats were cocultured with DP spheres	A core-shell structure, outer ASCs shell, and an inner DP core exhibited superior potential to HF formation compared to a mixed sphere of ADSCs with DP cells	PPAR*α* signal in ADSCs can induce the hair formation
Yang et al., 2016 [69]	Human/mice	C57BL/6 nude mice	Cocultured human ADSCs with LL-37 was topically applied daily on the mouse skin	The conditioned medium of ADSCs preactivated with LL-37 strongly promoted hair growth *in vivo*	LL-37 treatment significantly increased EGR-1 expression
Anderi et al., 2018 [122]	Human	Alopecia areata	Lipoaspiration, autologous ADSCs were injected into the scalp of the patient (4–4.7 × 10^6^ cells)	Increased hair growth and decreased pull test, 3 and 6 months after ADSCs	Significant variation was observed between men and women only for hair diameter, no differences with age

*Usage of Wharton's jelly and embryo stem cells*					
Yoo et al., 2010 [88]	Human	*In vitro* study	Cultivated umbilical stem cells with EGF, HGF, and NGF	Formation of aggregates similar to native DP in special media and reconstructed dermal papilla-like tissues	HGF is necessary in the differentiation step
Yoo et al., 2010 [89]	Human/mice	Athymic nude mice genetically mutated	Isolated and cultivated stem cells from bone marrow and umbilical cord, after obtaining a DP-forming medium, injected in skin of nude mice	Effect of inducing new HF in mice within 45 days	
Wu et al., 2012 [90]	Human embryo MSCs/mice	Nude mice genetically mutated	Three cultures: DP cells cocultured with hMSCs; DP cells cocultured with fibroblasts; hMSCs cultured single, next injected into skin of mice	In fibroblast injection to mice, no HF was found	The expression *in vivo* of HLA-I was confined to DP of the newly grown hair, and the survival time of hMSCs in mice is 1 month

ADSCs: adipose-derived stem cells; DP: dermal papilla; DSCs: dermal stem cells; EGF: epidermal growth factor; bFGF: basic fibroblast growth factor; HF: hair follicle; HGF: hepatocyte growth factor; HLA-I: human leucocyte antigen class I; hMSCs: human mesenchymal stem cells; IGFBP: insulin-like growth factor-binding protein; M-CSF: macrophage colony-stimulating factor; NGF: nerve growth factor; PGF: platelet-derived growth factor; VEGF: vascular endothelial growth factor.

**Table 3:** Current studies of stem cell use registered on ClinicalTrials.gov [122].

Number	Study	Kind of stem cells	Method	Conditions	Status	Trial institution/sponsor and country	NCT number and duration period
1	“Stem Cell Educator Therapy in Alopecia Areata”	Cord blood-derived multipotent stem cells (CB-SCs)	A closed loop system that circulates a patient's blood through a blood cell separator, briefly cocultures the patient's lymphocytes with adherent CB-SCs in vitro, and returns the educated lymphocytes (but not the CB-SCs) to the patient's circulation	Alopecia areata	UKN	The First Hospital of Hebei Medical University Shijiazhuang, Hebei, China	NCT01673789 2012-2013
2	“The Effect of Allogeneic Human Adipose Derived Stem Cell Component Extract on Androgenic Alopecia”	Allogeneic human ADSC component extract	Applying 1.2 g of allogeneic human adipose-derived stem cell component extract on their scalp for 16 weeks	Androgenic alopecia	Completed	Pusan National University Hospital, South Korea	NCT02594046 2015–2017
3	“Adipose Tissue Derived Stem Cell Based Hair Restoration Therapy for Androgenetic Alopecia”	Autologous MSC and human platelet-rich plasma	MSCs derived from adipose tissue with human platelet-rich plasma will be applied	Androgenic alopecia	Not yet recruiting	King Edward Medical University, Pakistan	NCT02865421
4	“Biocellular-Cellular Regenerative Treatment Scaring Alopecia and Alopecia Areata”	High-density platelet-rich plasma and adipose-derived tissue stromal vascular fraction (AD-tSVF)	Use of high-density platelet-rich plasma concentrates and cell-enriched emulsified adipose-derived tissue stromal vascular fraction (AD-tSVF) via intravenous infusion	Alopecia areata, scarring alopecia	Recruiting	Regeneris Medical, Global Alliance for Regenerative Medicine, Healeon Medical Inc., USA	NCT03078686 2017–2019
5	“AGA Biocellular Stem/Stromal Hair Regenerative Study”	Adipose-derived tissue stromal vascular fraction (AD-tSVF) and high-density platelet-rich plasma	Biocellular mixture of emulsified AD-tSVF and high-density platelet-rich plasma concentrate (HD-PRP) as compared with adipose-derived cell-enriched SVF (AD-cSVF)+AD-tSVF and HD-PRP using Healeon Centricyte 1000 system and intradermal injections	Androgenetic alopecia, female pattern hair loss	Recruiting	Healeon Medical Inc., Ministry of Health, Honduras Irvine, California, USA	NCT02849470 2016–2018
6	“Point-of-Care Adipose-derived Cells for Hair Growth”	Stromal vascular fraction (SVF) cells	A single injection into the scalp of autologous adipose-derived SVF cells	Androgenic alopecia	Recruiting	University of Florida Gainesville, Florida, USA	NCT02729415 2016-2017
